# The Influence of Motor Imagery on Postural Sway: Differential Effects of Type of Body Movement and Person Perspective

**DOI:** 10.5709/acp-0173-x

**Published:** 2015-09-30

**Authors:** John F. Stins, Iris K. Schneider, Sander L. Koole, Peter J. Beek

**Affiliations:** 1 Department of Human Movement Sciences, Faculty of Behaviour and Movement Sciences, MOVE Research Institute Amsterdam, VU University Amsterdam, Amsterdam, The Netherlands; 2 Department of Clinical Psychology, Faculty of Behaviour and Movement Sciences, VU University Amsterdam, Amsterdam, The Netherlands; 3 Department of Psychology, University of Southern Califonia, United States of America

**Keywords:** motor imagery, postural control, embodied cognition

## Abstract

The present study examined the differential effects of kinesthetic imagery (first person perspective) and visual imagery (third person perspective) on postural sway during quiet standing. Based on an embodied cognition perspective, the authors predicted that kinesthetic imagery would lead to activations in movement-relevant motor systems to a greater degree than visual imagery. This prediction was tested among 30 participants who imagined various motor activities from different visual perspectives while standing on a strain gauge plate. The results showed that kinesthetic imagery of lower body movements, but not of upper body movements, had clear effects on postural parameters (sway path length and frequency contents of sway). Visual imagery, in contrast, had no reliable effects on postural activity. We also found that postural effects were not affected by the vividness of imagery. The results suggest that during kinesthetic motor imagery participants partially simulated (re-activated) the imagined movements, leading to unintentional postural adjustments. These findings are consistent with an embodied cognition perspective on motor imagery.

## Introduction

People’s imagination allows them to picture themselves dancing, singing, sitting on a beach, or driving a car, even when in reality they are not. Imagining specific physical activities is referred to as motor imagery and can be defined as “the internal representation of an action without engaging in its physical execution” (p. 116, Di Rienzo, Collet, Hoyek, & Guillot, 2014). Motor imagery is often used for training purposes in sports (e.g., Reiser, Büsch, & Munzert, 2011), dance (Girón, McIsaac, & Nilsen, 2012), playing musical instruments (Lotze & Halsband, 2006), and neuro-rehabilitation (Ietswaart et al., 2011). As such, it seems important to learn more about the mechanisms underlying motor imagery.

According to theories of embodied cognition, conscious thought (such as engaging in mental imagery) consists of simulated interaction with the environment (e.g., Hesslow, 2002). In other words, thought is realized through sensorimotor simulations in the nervous system. Evidence for this (embodied) simulation hypothesis comes from studies demonstrating close parallels between simulated movements and actual movements, as evidenced by behavioral and neuroimaging studies (e.g., Mishra & Marmolejo-Ramos, 2010). With respect to motor imagery, it has been shown that mentally simulating a movement and performing the same movement recruits nearly identical neural (fronto-parietal) circuits (Hétu et al., 2013) (with the possible exception of the primary motor cortex).

Although these findings are consistent with an embodiment perspective, two important questions remain. First, what is the influence of the perspective taken in the imagery? When imagining a motor action or movement, it is possible to assume different perspectives. During *kinesthetic imagery*, one imagines the movement as if oneself is performing it. This type of imagery involves perceiving the movement through proprioceptive information—that is, through awareness of the limbs’ positions and velocities. Kinesthetic imagery is often also referred to as a first-person (or egocentric, or internal) perspective. In contrast, during *visual imagery* one imagines the movement as if one sees someone else performing it. This type of imagery thus involves visually perceiving the movement[Fn FN1]^1^, and is often referred to as a third-person (or allocentric, or external) perspective (e.g., Guillot et al., 2009). These different perspectives have differential effects as shown by brain imaging studies (e.g., Guillot et al., 2009; Sirigu & Duhamel, 2001) demonstrating that kinesthetic imagery and visual imagery represent dissociable neural processes. In addition, work on social judgments has suggested that person perspective modulates the effect of different embodiments (Macrae, Raj, Best, Christian, & Miles, 2013). More specifically, the more a mental simulation models the actual execution of an action, the more likely the simulation is to evoke the motor activity associated with the action. If so, kinesthetic imagery of a movement should lead to stronger activations of relevant motor systems than visual imagery. In line with this prediction, Moody and Gennari (2010) found that sentences describing actions involving various levels of physical effort induced corresponding changes in premotor regions.

A second question pertains to the effects of motor imagery on muscle activity and motor output. Some studies found subliminal changes in arm muscle activity while imagining manual activities. For example, Guillot et al. (2007) found that mentally imagining weight lifting led to subliminal changes in muscle activity, which were specific to different kinds of muscle contraction (cf. Bakker, Boschker, & Chung, 1996). However, the authors did not record kinematic changes—that is, objectively observable motor output. Given that the relationship between muscular activity and effector kinematics is highly non-linear, we tested whether motor imagery, via simulation in the related motor systems, can lead to unintended spatio-temporal changes in motor output. In the present study we asked whether motor imagery can impact on postural sway during quiet standing.

### Imagery and postural activity

Maintaining a quiet upright standing posture involves monitoring and controlling of the body orientation with respect to the gravity vector. This seemingly simple task involves the integration of visual, somatosensory, vestibular, and cortical inputs (Balasubramaniam & Wing, 2002), as well as making very rapid micro-postural adjustments in the face of external and internal perturbations. The resulting postural excursions or body sway, as evidenced in the body Center-of-Pressure (CoP) trajectories, displays remarkably complex dynamics. The CoP represents a complex output signal, emanating from various perceptual, attentional, cognitive and neurophysiological sources, which may themselves interact in a non-linear manner.

A number of recent studies have asked how imagining a movement leads to changes in body sway. We describe three studies that motivated the current experiment. Rodrigues et al. (2010) asked subjects, who were standing quietly in an upright posture, to imagine a sequence of bilateral plantarflexions—that is, rises on tiptoes. The main finding was that kinesthetic imagery of the movement sequence induced greater postural excursions than visual imagery. The authors speculated that the effectors involved in postural control received subliminal activation during kinesthetic imagery.

Grangeon, Guillot, and Collet (2011) likewise compared the effects of kinesthetic imagery and visual imagery on postural control. They contrasted two types of to-be-imagined activities, namely jumping, and performing a sequence of finger movements. Two main results emerged from the experiment. First, kinesthetic imagery led to overall more postural variability along all three body axes. Second, postural variability was higher when imagining jumping than when imagining finger movements. The authors suggested that during motor imagery muscle activity was not completely inhibited, which became manifest as greater postural activity.

More recently, Boulton and Mitra (2013) tested the effects of imagining discrete arm movements (reaches) on postural variability. Participants had to imagine making arm movements in the anterior posterior (i.e., front to back) or medio-lateral (side to side) direction. Crucially, participants were instructed to stand in one of two stance positions, namely feet closed together, or feet in a semi-tandem Romberg stance. This latter position is characterized by reduced postural stability in the medio-lateral (sideways) axis. One of the main findings was that postural instability increased in the direction of the to-be-imagined arm movements. In other words, motor imagery had direction-specific effects on postural sway. However, the Boulton and Mitra (2013) study did not manipulate participants’ perspective.

### Current Research and Hypotheses

In the present research, we had two major aims. Our first aim was to directly compare the effects of visual versus kinesthetic imagery on postural sway. To do so, we tested the physical effects of motor imagery (MI) of different motor activities involving different effectors (i.e., lower body vs. upper body) by measuring postural sway while participants stood on a force plate. Postural sway is related to postural control, which is predominantly a function of leg and hip muscles. Because imagining a movement involving the legs would lead to subliminal motor activation of associated muscles, this should lead to some degree of postural disruption—that is, more sway. Indeed, MI of upper body movements could likewise lead to motor activation of arm and shoulder muscles, but this should have less of an effect on postural sway. Thus, imagining lower body movements should affect participants’ postural sway, whereas upper body movements should not or significantly less so. We additionally predicted that kinesthetic imagery leads to greater postural effects than visual imagery, because kinesthetic imagery involves simulation of muscle activity and simulation of the associated sensory consequences (cf. Macrae et al., 2013).

Our second aim was to use richer imagery to increase the ecological validity of the findings, because we assume that in everyday life, people engage in MI that is richer than merely “tapping a finger.” To this end, in our study we tested imagery of cycling and jumping (i.e., involving the legs and lower body), and imagery of piano playing and waving (i.e., involving the arms and upper body). Additionally, we also tested MI of an activity that included little or no movement at all. To our knowledge, previous studies did not include such a neutral condition. Postural excursions recorded during a neutral condition can be used to compare the extent to which the different MI conditions contribute to enhanced postural sway.

## Method

### Participants

Thirty individuals (students at the VU University Amsterdam; 17 male, 13 female) who ranged in age from 18 to 36 years (mean age of 23 years; *SD* = 4 years) took part in the experiment. All participants signed an informed consent form prior to participation. None of the participants had visual or neuromotor impairments.

### Materials

CoP data were collected at 100 Hz for 30 s during each condition, using a custom made 1 m × 1 m strain gauge force plate. The force plate consisted of eight force sensors; four measuring forces in the z direction, and two each for the x and y directions. These eight signals were converted to forces (*F*_x_, *F*_y_, *F*_y_) from which moments (*M*_x_, *M*_y_, *M*_y_) were calculated. *M*_x_ and *M*_y_ were then used to calculate the point of application of the vertical force on the support surface—that is, the CoP.

### Procedure

Upon entering the lab, participants were told that they would be asked to imagine performing each of five activities (see below) both from a first-person perspective (kinesthetic imagery [KI]) and from a third-person perspective (visual imagery [VI]). To make sure that participants understood the difference between these two perspectives, the experimenter gave the example of swimming in a swimming pool.

After taking off their shoes, participants stepped onto the force plate, and the experimenter dimmed the lights. Participants were asked to adopt a quiet upright standing position, with the arms hanging relaxed alongside the body. There were five imagery scenarios: (1) gradually cycling uphill in a mountainous area, (2) bouncing on a trampoline positioned in a large garden, (3) waving at a friend who is walking at the opposite side of a street, (4) sitting while playing a piano in a quiet room, and (5) sitting quietly in a cinema theatre, waiting for the movie to begin. To manipulate upper and lower body related imagery, activities (1) and (2) were designed to relate to movements involving the legs and lower body, whereas activities (3) and (4) relate to movements involving the arms and hands and the upper body. The fifth activity involved no discernible motor activity, and was considered neutral. Together, these five scenarios represented motor activities that were relatively easy to imagine, and that could be imagined without actual prior experience with the activity. For example, someone who has never played the piano in their lifetime can still imagine the bodily movements and postures associated with this activity.

The imagery scenarios were presented in random order within two blocks, one for KI and one for VI. The blocks were counterbalanced between participants. Thus, each participant completed 10 trials in total. At the start of each trial, the imagery script was read aloud by the experimenter, after which the experimenter started the 30 s postural data recording session (cf. Grangeon et al., 2011). During each imagery episode participants were not allowed to move or to speak.

At the end of each trial (indicated by the experimenter), participants verbally provided a vividness rating on how well they were able to imagine that particular motor activity for the duration of the trial. Values could range from 1 (*no imagery at all*) to 6 (*very clear and vivid imagery*; cf. Grangeon et al., 2011). The rating procedure was verbally explained at the start of the experiment. Each reported value was written down by the experimenter (and later entered into the computer), after which the next trial started.

### Design and analysis

Prior to all analyses high frequency components were removed from the time series by applying a 15 Hz low pass Butterworth filter. CoP excursions were analyzed using two broad classes of parameters, related to (1) the amount of sway, and (2) the frequency contents of sway.

#### COP: Amount of sway

Theoretically, when an individual is standing completely motionless, postural excursions in any direction will be zero. However, biological systems are always subject to small internal and external perturbations, meaning they are inherently noisy, so there will always be some amount of motor output variability, such as postural fluctuations. Postural oscillations often occur involuntarily, even without an individual’s explicit knowledge or awareness. Although individuals may have the experience of completely standing still, sensitive equipment may still pick up subtle task-induced postural fluctuations. So, when an individual is pivoting around the ankle (as happens in normal quiet stance), postural excursions will be greater than zero. More extreme postural instability, as for example in pathology (e.g., Stins, Ledebt, Emck, Dokkum, & Beek, 2009) or when drunk, is characterized by larger amounts of sway, and may be a precursor to a fall.

*Amount of sway* was quantified using the following measures:

 1. *SD* [CoP AP]; the within-trial standard deviations of the CoP in the antero-posterior (AP) direction. This is related to postural excursions in the fore-aft direction. 

 2. *SD* [CoP ML]; the within-trial standard deviations of the CoP in the medio-lateral (ML) direction. This is related to postural excursions in the left-right direction. 

 3. *SD* [vertical force]; the within-trial standard deviations of the force exerted in the vertical (up down) direction. This happens for example when a participant were to repeatedly flex the knees (lowering the center of mass) and then extend the knee (raising the center of mass)[Fn FN2]^2^. 

 4. Sway path length (SPL). This is the summed length of postural excursions in the AP-ML plane. SPL was calculated by consecutively summing the distances between adjacent points of the CoP trace. 

These four values are identical to the ones reported by Grangeon et al. (2011).

#### CoP: Frequency contents of sway

Postural excursions are not purely random, but exhibit characteristic frequencies, which for biomechanical reasons are predominantly related to stiffness of the ankle joint, and the length of the body (see Winter, 1995, for details). A commonly used metric in posturography is the mean power frequency (MPF), which is an estimate of the average frequency contained within the power spectrum (e.g., Carpenter, Frank, Silcher, & Peysar, 2001). MPF was calculated separately for sway in the anterior-posterior (MPF AP) and medio-lateral (MPF ML) directions. These values complement values related to the amount of sway, as they provide insight into the manner in which balance is regulated.

#### Statistical analysis

Prior to all analyses, the values of the two upper body activities and of the two lower body activities were averaged. Each of the six postural parameters described above (related to amount of sway and frequency contents of sway), as well as the vividness ratings were then submitted to separate repeated-measures analyses of variance (ANOVA), with activity type (upper body, lower body, and rest) and imagery type (KI vs. VI) as factors. Alpha-level was set at 0.05. Effect sizes of the ANOVA are reported as partial eta-squared (η^2^_p_), and effect sizes of the simple contrasts are reported as Cohen’s *d* (see Lakens, 2013). For benchmarks to define small, medium, and large effects see Cohen (1988). We also report 95% confidence intervals (CI) for the mean difference between conditions. Note that, similar to most posturographic studies, we used parametric tests to analyze the variables. This choice was motivated by the consideration that (a) CoP values are measured on a continuous scale, similar to, for example, reaction times, and (b) ANOVA is generally robust against violations of normality (e.g., Schmider, Ziegler, Danay, Beyer, & Bühner, 2010).

## Results

The data of one (male) participant were not analyzed due to technical difficulties. Mean values of all parameters are reported in Table 1.

**Table 1.** Mean Values of All Parameters

	KI upper body	KI lower body	KI rest	VI upper body	VI lower body	VI rest
Vividness	4,3 (1,1)	4,6 (1,0)	4,5 (1,2)	3,9 (1,0)	4,3 (1,1)	3,9 (1,2)
Amount of sway *SD* [CoP AP] (mm)	4,8 (2,2)	4,9 (1,5)	4,8 (1,6)	4,8 (1,6)	4,9 (1,7)	4,6 (1,6)
*SD* [CoP ML] (mm)	2,0 (1,0)	2,2 (1,0)	1,8 (1,0)	2,0 (1,1)	2,0 (0,9)	1,9 (0,8)
*SD* [vertical force] (N)	0,91 (0,23)	0,95 (0,26)	0,94 (0,24)	0,92 (0,25)	0,93 (0,25)	0,92 (0,25)
SPL (mm)	673 (92)	693 (101)	666 (88)	671 (107)	673 (99)	673 (104)
Frequency of sway MPF AP (Hz)	0,35 (0,18)	0,35 (0,13)	0,30 (0,14)	0,32 (0,15)	0,35 (0,15)	0,34 (0,17)
MPF ML (Hz)	1,06 (0,83)	0,80 (0,46)	1,01 (0,72)	0,90 (0,52)	0,95 (0,63)	0,97 (0,63)

*Note*. *SD* = standard deviation (values in parentheses); KI = kinesthetic imagery; VI = visual imagery; CoP = center of pressure; AP = anterio-posterior; ML = medio-lateral; MPF = mean power frequency.

### Amount of sway

**Figure 1.**
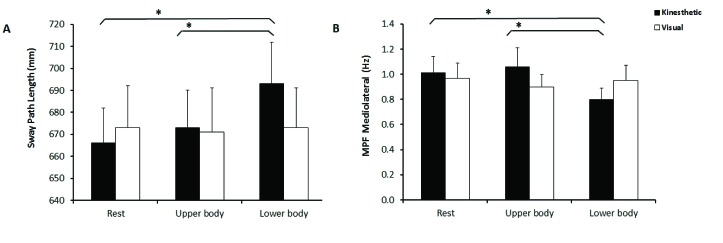
A: Sway path length (mm) for the six conditions. Significant (*p* < .05) contrasts between conditions are denoted with an asterisk (*). Error bars denote standard errors of the mean. B: Mean power frequency (Hz) for the six conditions. Significant (*p* < .05) contrasts between conditions are denoted with an asterisk (*). Error bars denote standard errors of the mean.

Only the main effect of activity type for the sway path length was significant, *F*(2, 56) = 3.64, *p* < .05, η^2^_p_ = .12, which was qualified by the predicted interaction between activity type and imagery type, *F*(2, 56) = 4.13, *p* < .05, η^2^_p_ = .13 (see Figure 1A). Planned comparisons (paired *t*-tests) revealed that SPL of KI of lower body movements was significantly larger than KI of upper body movements, *t*(28) = 2.83, *p* = .009, *d* = 0.53, 95% CI [5.47, 34.21]. Also, SPL of KI of lower body movements was higher than KI of resting, *t*(28) = 3.36, *p* = .002, *d* = 0.62, 95% CI [10.50, 43.38]. The same contrasts for VI were not significant. The other three variables (*SD* [CoP AP], *SD* [CoP ML], and *SD* [vertical force]) yielded no significant effects.

### Frequency contents of sway

For the MPF ML, there was a significant main effect of activity type, *F*(2, 56) = 3.36, *p* < .05, η^2^_p_ = .11. We performed separate ANOVAs for KI and VI (with activity type as within-subject factors). These analyses revealed that there was no effect of activity type for VI, whereas the effect was significant for KI, *F*(2, 56) = 5.36, *p* < .01, η^2^_p_ = .16. Planned comparisons (paired *t*-tests) revealed that MPF ML of KI of lower body movements was significantly lower than KI of upper body movements, *t*(28) = 2.69, *p* = .012, *d* = 0.50, 95% CI [0.06, 0.46]. Also, MPF ML of KI of lower body movements was lower than the KI resting condition, *t*(28) = 2.95, *p* = .006, *d* = 0.55, 95% CI [0.07, 0.36]. No effects involving MPF AP were significant. Means are displayed in Figure 1B.

### Vividness

For the vividness ratings we found that KI (mean value: 4.5; *SD* = 0.79) led to higher ratings than VI (4.0; *SD* = 0.83), *F*(1, 28) = 12.23, *p* < .01, η^2^_p_ = .30. No effects involving activity type were significant. To examine the degree to which postural sway was influenced by imagery vividness, we correlated (using Spearman’s *r*) the average vividness ratings with overall (i.e., averaged over conditions) posturographic outcome measures. Neither the SPL (*r* = .048, *p* = .806) nor the MPF ML (*r* = .032, *p* = .868) correlated significantly with vividness, indicating that postural performance was unaffected by variations in MI vividness.

## Discussion

The aim of the present study was to test whether MI would lead to effector specific postural adjustment, depending on person perspective. If so, this would lend support for the embodied nature of motor representations and corresponding theoretical notions. To this end, we examined the differential effects of KI (first person perspective) and VI (third person perspective) on postural sway during quiet standing. Crucially, we compared MI of upper body movements, lower body movements, and a resting situation.

First, we found elevated sway path lengths when participants were imagining lower body activities (trampoline bouncing and cycling) performed from a first person perspective KI, relative to other activities. The third person perspective VI, in contrast, yielded no differential effects on postural activity. This finding is in agreement with the findings of Rodrigues et al. (2010) and Grangeon et al. (2011), and suggests that MI of this type induced enhanced postural sway, which may index postural instability. Note that we found an effect predominantly in sway path length, and not in postural variability along the *x*, *y*, or *z*-axis. Grangeon et al. (2011), in contrast, found significant effects predominantly for their variability measures along all three axes. Although, in general, both findings point to motor activation, the origin of these differences remains unclear.

Second, we found that KI of lower body movements was characterized by low frequencies of sway in the medio-lateral direction. According to Balasubramaniam and Wing (2002) excursions of the CoP along the anterior-posterior axis reflect predominantly plantarflexion and dorsiflexion around the ankle joint, whereas excursions along the medio-lateral axis reflect abduction and adduction about the hip joint. Thus, KI of lower body activities resulted in slow sideways postural oscillations. Given that the to-be-imagined lower body activities involve bilateral simultaneous leg movements (trampoline bouncing) and bilateral alternating leg movements (cycling), our analysis suggests that MI of rhythmic movements also had a clear effect on the frequency contents of sway. This is a novel finding, as the literature thus far has mainly focused on the amount of sway (*SD* and SPL) and not its temporal structure. Our analysis suggests that postural effects of MI might even be more specific than thus far anticipated. Future studies should explore how tight the coupling between MI and postural sway is. One testable hypothesis is that changes in the level of effort of imagined motor activity should lead to corresponding changes in postural sway. Moody and Gennari (2010) found that levels of physical effort implied in verbal material led to corresponding neural changes. Bakker et al. (1996) found that imagining lifting heavy weights led to greater changes in EMG activity compared to lighter weights. Based on these findings we predict that, for example, imagining a bicycle ride involving a steep and effortful ascent will lead to greater postural excursions than imaging a leisurely bicycle ride through the Dutch landscape.

A possible explanation for our findings—and those of others—is that during MI participants made subliminal and unintentional postural adjustments. That is, the mental simulation of the movements recruited similar networks as during actual action execution. The brain imaging study of Guillot et al. (2009) revealed that VI recruited predominantly visual cortical areas, whereas KI resulted in activity in motor-related areas, such as the basal ganglia and cerebellum. Postural activity during MI is thus thought to result from incomplete motor inhibition. This is in line with theorizing on grounded cognition that predicts that imagining a certain movement involves simulation (or re-activation) of previous experiences with that movement, reactivating (partly) the motor areas associated at the time (e.g. Barsalou, 1999, 2008). We are aware of one study that jointly examined postural sway and postural muscular activity. Lemos, Rodrigues, and Vargas (2014) reanalyzed the electromyographic (EMG) data collected by Rodrigues et al. (2010). Two main findings emerged. First, even though KI modulated postural sway (see the Introduction), there was no net change in mean EMG amplitude. Second, cross-correlation analysis of the EMG-CoP time series revealed a stronger EMG-CoP association during KI. According to the authors, this latter finding might have been due to changes in motoneuron excitability, which modulates the temporal coupling (synchronization) between muscle activity and sway.

It could also be argued that our findings represent dual-tasking effects. Our study involved the combined execution of a postural task (quiet standing) and a cognitive task (MI). The literature suggests that maintaining static balance is to a large extent automatized (hence, requiring few attentional resources), but at the same time sensitive to cognitive activity. Especially individuals with balance problems, such as the elderly, may find it difficult to combine postural tasks with cognitive tasks (for a review see Frazier & Mitra, 2008). There is evidence that when the attentional demands of a cognitive task increase, this leads to a concurrent increase in postural sway (e.g., Pellecchia, 2003). Thus, it could have been the case that the critical condition (KI of lower body movements) was the most cognitively demanding form of MI, leading to a concurrent increase in sway. Although we cannot rule out this possibility, we find it unlikely because the vividness ratings showed no differential effects of imagery activity. That is, vividness was equally high for MI of upper body movements, lower body movements and resting, although overall vividness of KI was higher than vividness of VI.

As a third possibility, it could be that the postural adjustments in fact facilitate information processing during MI. That is, the observed postural activity could reflect attempts of the actor to perform the MI task as requested, so that postural activity is in fact adaptive to the task at hand, and not merely reactive. In a similar vein, Lorey et al. (2009) argued that MI is a “profound body-based simulation process that uses the motor system as a substrate” (p. 234). There is converging evidence that the state of the motor system can shape information processing. For example, it has been shown that motoric syndromes, such as Parkinson´s disease (PD), negatively impact on the ability to process action-related concepts, such as verbs but not concrete nouns (e.g., Boulenger et al., 2008; Cardona et al., 2014; Geboers & Stins, 2014). The review of Di Rienzo et al. (2014) convincingly showed that various neurologic disorders, including PD, impacted on various aspects of MI. Future work using unaffected individuals may investigate the embodiment of MI further by blocking motor activity during imagery.

One limitation related to this study, and similar studies, is that the experiment critically revolves around participants’ ability and willingness to perform the requested MI, and subsequently their ability to reliably report vividness via self report. Although in general our participants reported being able to follow instructions, we have no independent evidence that they actually did. Despite our clear-cut and theoretically meaningful results, future studies could use different MI instructions that allow independent measures of MI performance, such as speed of mental rotation of a picture of a hand, or a comparison between actual and imagined movements (e.g., Grangeon et al., 2011).

## Conclusions

In sum, this work shows that MI has effector specific influences on postural sway, and that these influences are dependent on the adopted person perspective. The findings are in agreement with current theorizing on the embodied nature of mental activity.

## Footnotes

1 Depending on instructions visual imagery can involve imagery of the own bodily movements, or the movements of someone else.

## Footnotes

2 Note that this value corresponds to “CoP vertical axis’” in the Grangeon et al. (2011) study. However, the CoP by definition involves the point of application of the ground reaction force vector, and therefore has no vertical component. The centre of mass (CoM), in contrast, does have a vertical component, and subtle up-down movements of the CoM will result in elevated values of our parameter.
